# Multiple myeloma in the real-world practice: clinical and prognostic indeces – single center experience

**DOI:** 10.3389/fonc.2026.1869023

**Published:** 2026-07-29

**Authors:** Natalija Kecman, Aleksandra Sretenovic, Marko Mitrovic, Zoran Bukumiric, Igor Tanović, Nikola Vukosavljevic, Jelica Jovanovic, Marija Dencic Fekete, Jelena Bila

**Affiliations:** 1 Clinic of Hematology, University Clinical Center of Serbia, Belgrade, Serbia; 2 Medical Faculty, University of Belgrade, Belgrade, Serbia; 3 Institute of Statistics and Bioinformatics, Medical Faculty, University of Belgrade, Belgrade, Serbia; 4 Department for Hemato-oncology, University Clinical Hospital Center “Zvezdara”, Belgrade, Serbia; 5 Laboratory for Molecular Pathology, Institute for Pathology, Medical Faculty, University of Belgrade, Belgrade, Serbia

**Keywords:** IMWG criteria, multiple myeloma, prognosis, R2-ISS scoring system, real world data (RWD)

## Abstract

**Background:**

The Second Revision of the International Staging System (R2-ISS) is an prognostic model established in newly diagnosed multiple myeloma (NDMM) patients as a marker of disease-related biological risk. Still, the course of disease is influenced by patient-related factors as well. The International Myeloma Working Group (IMWG) frailty score provides a standardized assessment of patient fitness and may complement biological risk stratification. The aim of study was to analyze prognostic significance of R2-ISS scoring system and IMWG frailty status in NDMM patients (pts) within RWE settings of the routine clinical practice.

**Methods:**

Analyzed group included 371 NDMM patients (pts, 184 males/187 females, median age 62.9 years, range 36-88) diagnosed between 2018 and 2025. Patients were stratified at the time of diagnosis according to the ISS, R-ISS, R2-ISS, and the original IMWG frailty score. Treatment response and follow-up were assessed according to the IMWG response criteria and EHA-EMN recommendations. Differences in progression-free survival (PFS) and overall survival (OS) were assessed using the Kaplan-Meier method and log-rank test. Independent OS predictors were identified using the Cox proportional hazards regression model.

**Results:**

Treatment response (≥PR) was achieved in 275 of 340 evaluable patients (80.9%). Increasing R2-ISS stage was associated with a lower probability of achieving treatment response (p=0.027), while frailty independently correlated with inferior response rates (p<0.001). Both PFS (p < 0.001) and OS (p < 0.001) was significantly shorter in patients with higher R2-ISS stages. In accordance to the IMWG frailty score, fit patients had significantly durable PFS (p<0.001) and the OS (p <0.001). Importantly, the composite classification integrating R2-ISS and IMWG frailty score provided additional discrimination of OS (p<0.001). Multivariate model for OS pointed out most significant impact of frailty status (p<0.001), followed by treatment response categories (p<0.001), and R2-ISS (p=0.009).

**Conclusion:**

R2-ISS and IMWG frailty score provide complementary prognostic information in NDMM. Their combined assessment improves risk stratification beyond disease-related factors alone and may facilitate more individualized treatment planning in the routine clinical practice.

## Highlights

- The R2-ISS and IMWG frailty score provide complementary prognostic information in newly diagnosed multiple myeloma. Their combined assessment improves risk stratification and may support more individualized treatment decisions in routine clinical practice.

## Introduction

Multiple myeloma (MM) is a biologically heterogeneous plasma cell malignancy characterized by substantial variability in treatment response and survival outcomes. Accurate identification of patients (pts) with high-risk disease is essential for prognostic assessment, treatment selection, and optimization of therapeutic strategies. Over the past decades, several prognostic models have been developed such as International Staging System (ISS), the Revised International Staging System (R-ISS), followed by the Second Revision of the International Staging System (R2-ISS), which incorporates ISS stage, serum lactate dehydrogenase (LDH), del(17p), t(4;14), and gain/amplification of 1q21 (1–3). The recently published consensus of International Myeloma Society/International Myeloma Working Group (IMS/IMWG) recommendations on high-risk MM have further refined risk assessment of MM patients by combining findings of molecular genetics including:1) del17p in >20% of clonal plasma cells and/or TP53, 2) IgH translocations [t(4;14), t(14;16), or t(14;20)], along with gain/amplification of chromosome 1q21 and/or del1p32, 3) monoallelic del 1p32 along with +1q32 or biallelic del1p32; with 4) concentration of β2 microglobulin ≥5.5mg/l in the case of normal creatinine level (<1.2mg/l). According to these recommendations, high-risk disease is defined by the presence of adverse biological characteristics associated with significantly inferior progression-free and overall survival. Although this consensus represents the most comprehensive contemporary framework for risk stratification, its routine implementation may be limited by the availability of advanced molecular diagnostic techniques. However, in accordance to the European Myeloma Network (EMN) Report, patient-specific biomarkers such as validated frailty scores, significantly affect the course of disease and should be explored in combination with R2-ISS (3, 4).

Regarding patient-related factors with impact on the course of disease, IMWG developed the frailty score, which integrates age, comorbidity burden, and functional status to identify 3 group of patients defined as fit, intermediate-fit, or frail. Similarly to the findings presented in the EMN Report, the authors of the IMWG frailty score highlights the necessity of incorporating both disease- and patient-related prognostic factors in the treatment decision-making (3, 5).

Several real-world studies have validated the prognostic performance of the R2-ISS across diverse patient populations (6, 7). Report from the Balkan Myeloma Study Group (BMSG) pointed out that R2-ISS stratification in the real-world settings better determinates patients with poor prognosis in comparison to the R-ISS staging system (8). However, Uesugi Yuka on behalf of the Japanese group of authors, emphasized the importance of including the patient’s age in order to predict survival outcome accurately (9).

Still, the integration of the R2-ISS stratification and IMWG frailty assessment, with impact on the course of disease and survival outcome, retain clinical interest in the settings of the routine clinical practice.

The aim of study was to analyze both of prognostic significance of R2-ISS scoring system and IMWG frailty status in newly diagnosed MM (NDMM) patients within RWE settings of the routine clinical practice.

## Materials and methods

### Patients

This retrospective study included 371 NDMM patients diagnosed and treated at the Department for Multiple Myeloma, Clinic of Hematology, University Clinical Center of Serbia, between 2018 and 2025. The median age was 62.9 years (range 36-88yrs), with slight predominance of female patients (50.4%). The most frequent myeloma subtype was IgG (227pts, 61.2%), followed by IgA (72pts, 19.4%), Bence Jones (BJ, 62pts, 16.7%), and IgM (1pt, 0.3%). Biclonal M protein was detected in 9pts (2.4%). Clinical stage (Durie&Salmon, CS) III had 318pts (85.7%), CS II 35pts (9.4%); and symptomatic CS I was found in 18pts (4.9%) (10). Renal impairment was present in 96pts (25.9%), extramedullary disease (EMD) in 63pts (16.9%). Baseline demographic, clinical, laboratory, and disease-related characteristics are summarized in **Table 1**.

**Table 1:** The patients’ clinical characteristics.

Clinical characteristics	N (%)
Gender	
Male	184 (49.6)
Female	187 (50.4)
Type of MM	
IgG	227 (61.2)
IgA	72 (19.4)
BJ	62 (16.7)
IgM	1 (0.3)
Biclonal	9 (2.4)
Type of light chain	
Kappa	241 (65)
Lambda	124 (33.4)
Biclonal	6 (1.6)
CS (Durie-Salmon)	
I	18 (4.9)
II	35 (9.4)
III	318 (85.7)
Renal impairment	96 (25.9)
EMD	63 (16.9)
Transplant eligible	203 (54.7)
Transplant ineligible	168 (45.2)

Frailty assessment was performed using the original IMWG frailty score calculator (5). Based on the total score, patients were classified as fit, intermediate-fit, or frail. These data are shown in **Table 2**.

**Table 2:** Frequency of patients using IMWG frailty score.

Category	N (%)
Fit	114/371 (30.7)
Intermediate-fit	156/371 (42)
Frail	101/371 (27.1)

Patients were treated with bortezomib-based triplet regimens (Bz + IMiDs). High-dose therapy with melphalan followed by autologous stem cell transplantation (ASCT) was conducted in 120/203pts (59.1%) initially characterized as a transplant candidates. The treatment approach was based on the international recommendations valid at the time of diagnosis and the patient’s characteristics as previously described: 1) age ≤70 years and transplant eligibility; 2) staging in accordance with the R-ISS and R2-ISS score, and/or presence of double-triple hit myeloma; and 3) IMWG frailty score (1–3, 5, 11–15). The escalation after the second cycle of treatment from Bz-based doublets to Bz+IMiDs triplets was performed in 91 frail patients (24.5%) after recovery to intermediate-fit status. Treatment response and follow-up were assessed according to the IMWG response criteria and EHA-EMN recommendations (15, 16). Patients who died within the first 6 months after diagnosis were excluded from the response analysis.

Following the acquisition of patients’ written informed consent, clinical data were systematically collected. The study was conducted in accordance with the ethical standards of the Declaration of Helsinki and the principles of Good Clinical Practice. Ethical approval was obtained from the Institutional Board of the Hematology Clinic and the Ethics Committee of the University Clinical Center of Serbia.

### Methods

In all NDMM patients, specific chromosomal abnormalities (CAs: t(4;14); del(17p); t(14;16), +1q21; amp1q21) were examined at the smears of plasma cells isolated from the bone marrow aspirates and purified with CD138 microbeads with purity level of 90%. Interphase fluorescence *in-situ* hybridization (iFISH) was performed as previously described, according to ESMO, EHA-ESMO and EHA-EMN Guidelines for diagnosis, treatment and follow-up (13–15). The minimum number of nuclei scored was 200 per case. The deletion 1p analysis was performed using the iFISH probe targeting the 1p32 region.

Missing cytogenetic results were not interpreted as negative, and they were not included in statistical analysis. The number of patients with available data for each component is presented in the revised **Table 3**. Due to retrospective character of this real-world study, complete cytogenetic testing was not conducted in all of the patients, and this limitation was considered when interpreting the results.

**Table 3:** Frequency of cytogenetic abnormalities (CA) and frequency of double/triple hit MM.

CA	N (%)
del 17p	66/349 (18.9)
t (4;14)	8/349 (2.3)
t (14;16)	3/348 (0.9)
gain 1q	66/349 (18.9)
amp 1q	50/349 (14.3)
del 1p	35/349 (10)
Double/triple-hit MM	24/348 (6.9)

For the ISS staging, serum β2 microglobulin and serum albumin levels at diagnosis were examined (1). R-ISS was calculated using ISS score, LDH level and the presence of high-risk cytogenetic abnormalities, including del(17p), t(4;14), and t(14;16) (2). R2-ISS was calculated according to the original R2-ISS model, incorporating ISS stage, LDH level, del(17p), t(4;14), and 1q abnormalities (3). Elevated LDH was defined as a value above the institutional upper limit of normal (ULN, 418 U/L). Cytogenetic abnormalities were considered positive when detected in ≥20% of analyzed nuclei. Gain (1q) was defined as the presence of three copies of 1q21, whereas amplification of 1q [amp(1q)] was defined as the presence of four or more copies. Double-hit MM was defined as the presence of any two high-risk cytogenetic abnormalities, while triple-hit MM was defined as the presence of any three high-risk abnormalities among del(17p), t(4;14), t(14;16), del(1p) and 1q21 gain/amplification (11).

The stratification of MM patients in analyzed group according to ISS, R-ISS, and R2-ISS staging systems is shown in **Table 4**.

**Table 4:** The stratification of patients using different scoring systems.

Scoring system	N (%)
ISS	
1	102 (27.5)
2	102 (27.5)
3	167 (45)
R-ISS	
1	84 (22.6)
2	239 (64.4)
3	48 (12.9)
R2-ISS	
Low risk, R2-ISS I	65/349 (18.6)
Intermediate-low, R2-ISS II	83/349 (23.8)
Intermediate-high, R2-ISS III	144/349 (41.3)
High-risk, R2-ISS IV	57/349 (16.3)

### Statistical analysis

Patients’ clinical characteristics were summarized using descriptive statistics. Depending on the type of variable and the distribution of continuous data, results were presented as frequencies and percentages (n, %) or as the median (minimum–maximum). Inferential statistical analyses were performed using the Mann–Whitney U test. All statistical tests were two-sided, and statistical significance was defined as p < 0.05. Time-to-event outcomes were analyzed using the Kaplan–Meier method. Differences in progression-free survival (PFS) and overall survival (OS) according to the R2-ISS stage and IMWG frailty score were assessed using the log-rank test. Independent predictors of the OS were identified using the Cox proportional hazards regression model, and results were reported as hazard ratios (HRs) with 95% confidence intervals (95% CIs). All analyses were performed using IBM SPSS Statistics version 31 (IBM Corp., Armonk, NY, USA) and the R statistical environment (R Core Team, 2025).

## Results

A favorable treatment response (CR, VGPR, or PR) according to the IMWG response criteria was achieved in 275 of 340 evaluable patients (80.9%), including 30 patients (8.8%) who achieved CR, 131 (38.5%) VGPR, and 114 (33.5%) PR (16). Patients who died within the first 6 months after diagnosis were excluded from the response analysis. Significant association was observed between R2-ISS score and achievement of a treatment response. Patients with lower R2-ISS scores were more likely to achieve a treatment response (Mann–Whitney U = 6343; Z = −2.209; p = 0.027).

In addition, significant association was observed between IMWG frailty categories and achievement of treatment response. Fit patients demonstrated the highest rate of achieving treatment response (90.4%), followed by intermediate (80.1%) and frail patients (68.8%). In this view, increasing frailty status was associated with a lower probability of achieving treatment response (Mann–Whitney U = 6437.5; Z = −3.760; p < 0.001).

**Figure 1:**
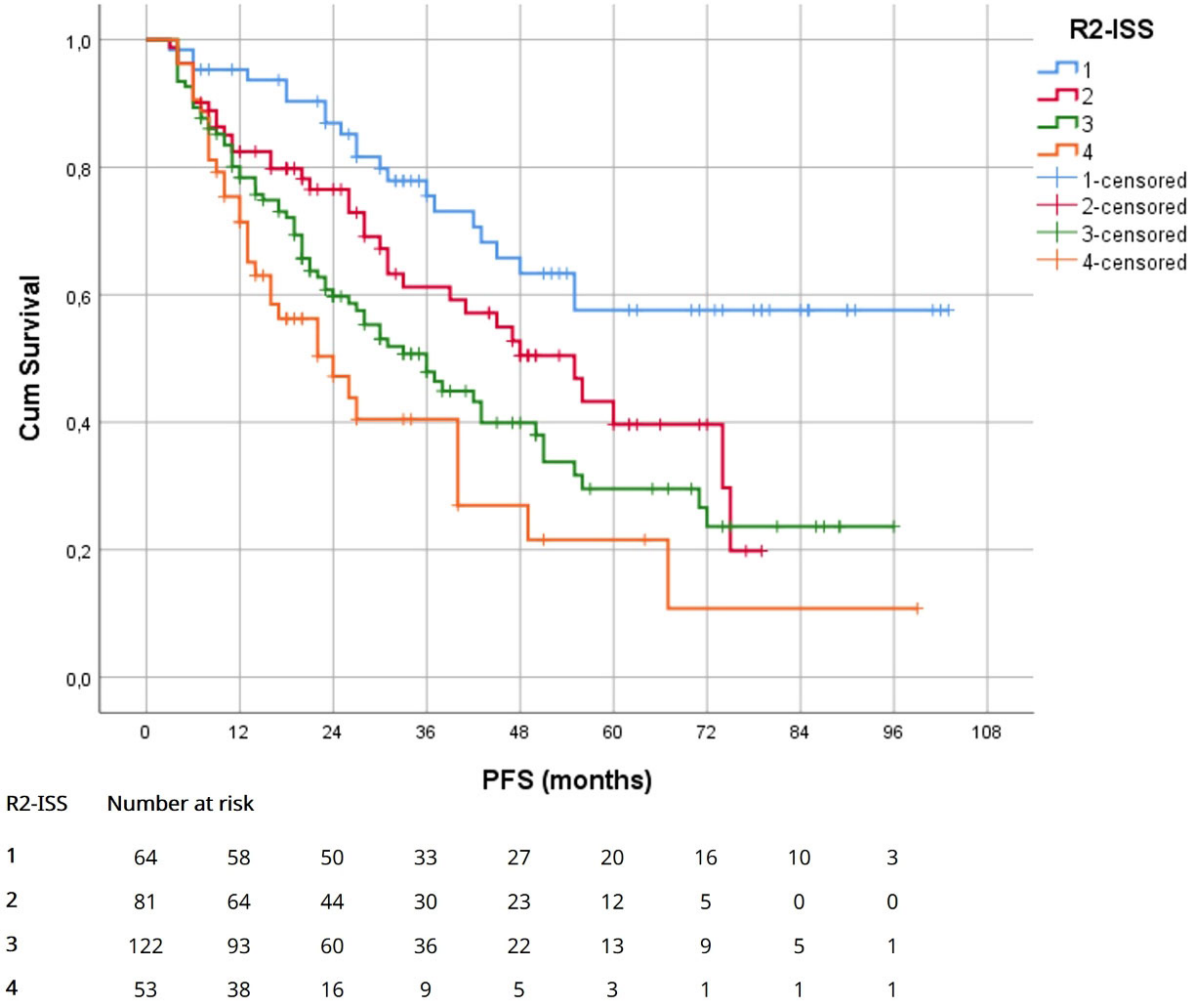
Kaplan–Meier curves of PFS stratified by R2-ISS groups.

Regarding survival analyses, PFS was significantly shorter in patients with higher R2-ISS stages (log-rank test: χ² = 27.424, df = 3, p < 0.001), confirming the prognostic discrimination provided by the R2-ISS classification (**Figure 1**).

**Figure 2:**
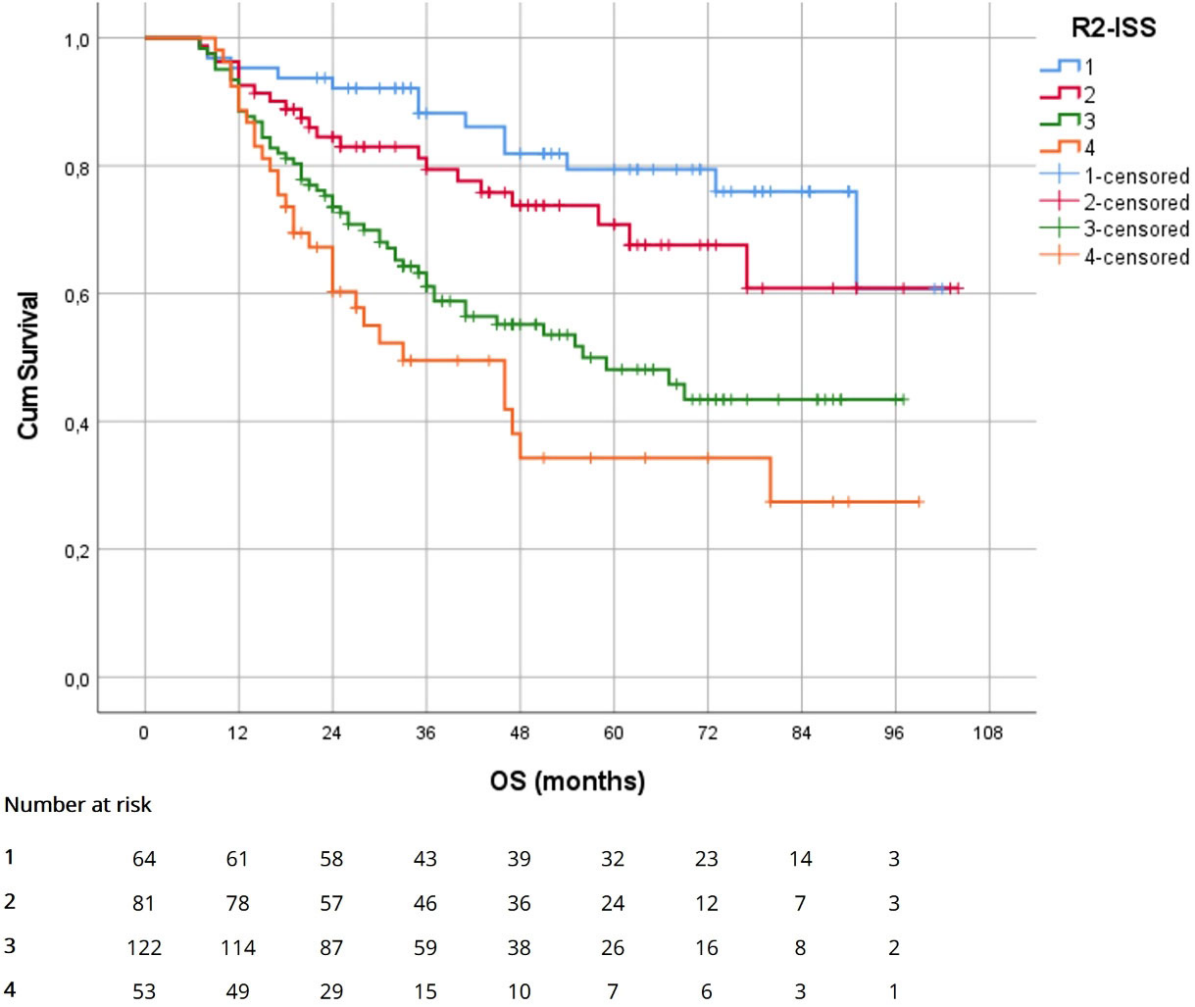
Kaplan–Meier curves of OS stratified by R2-ISS groups.

The Kaplan–Meier OS analysis according to the R2-ISS classification demonstrated progressively shorter survival with increasing R2-ISS stage. The estimated mean OS was 85.5 months in R2-ISS group 1, 78.5 months in group 2, 59.4 months in group 3, and 49.4 months in group 4. The median OS was 56 months in R2-ISS group 3 and 33 months in group 4, while it was not reached in groups 1 and 2 during the follow-up period. The overall mean survival for the entire cohort was 69.6 months (95% CI: 64.8–74.3), with a median OS of 91 months. Survival distributions differed significantly between R2-ISS groups (log-rank test: χ² = 30.412; df = 3; p < 0.001), indicating the prognostic value of the R2-ISS classification in risk stratification (**Figure 2**).

**Figure 3:**
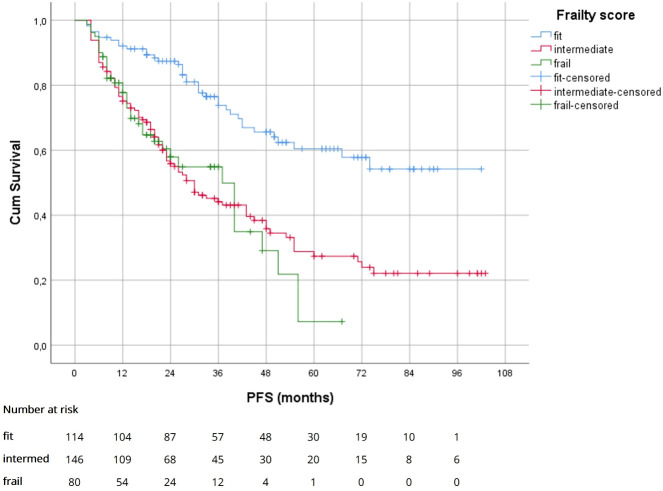
Kaplan–Meier PFS curves according to the IMWG frailty score.

In accordance to the IMWG frailty score, fit patients had significantly durable PFS in comparison to the intermediate-fit and frail patients (log-rank test: χ² = 32,020, df = 2; p < 0.001) (**Figure 3**).

**Figure 4:**
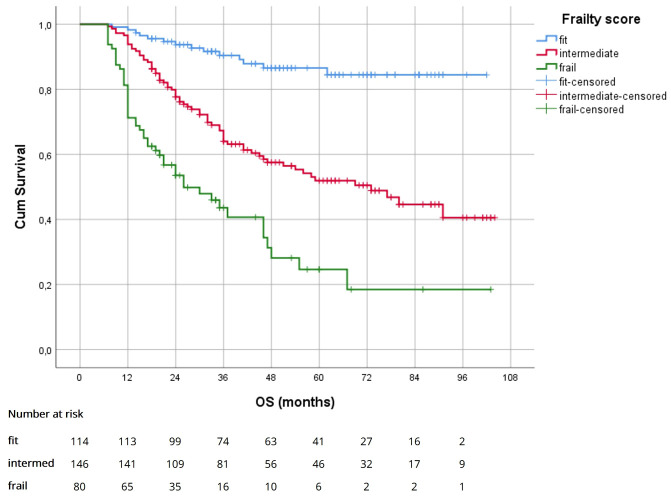
Kaplan–Meier OS curves according to the IMWG frailty score.

Regarding the survival analysis between the IMWG Frailty categories, the Kaplan–Meier OS analysis demonstrated significant differences (log-rank test: χ² = 72.682, df = 2; p < 0.001), indicating the prognostic value of frailty assessment for survival stratification (**Figure 4**). The estimated mean OS was 91.1 months in fit patients, 65.8 months in intermediate patients, and 41.2 months in frail patients. Median OS was not reached in the fit group, while it was 73 months and 26 months in the intermediate and frail groups, respectively.

**Figure 5:**
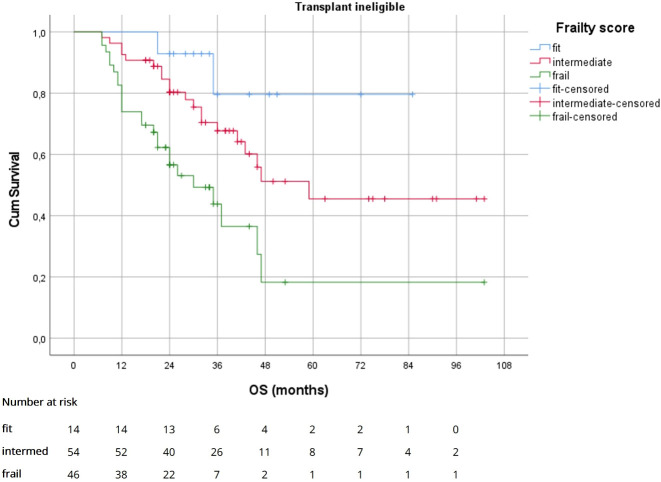
Kaplan–Meier OS sub-analysis according to the IMWG frailty score in transplant ineligible patients.

Furthermore, sub-analysis focusing on IMWG frailty score in transplant ineligible patients, confirmed significant differences in the OS according to the IMWG Frailty categories in this subgroup. The estimated mean OS was 73.8 months in fit patients, 64.9 months in intermediate patients, and 40.2 months in frail patients. Median OS was not reached in the fit group, while it was 59 months and 30 months in the intermediate and frail groups, respectively. Survival distributions differed significantly between Frailty score groups (log-rank test: χ² =14.583; df = 2; p = 0.001), indicating the prognostic value of frailty assessment for survival stratification in transplant ineligible patients (**Figure 5**).

**Figure 6:**
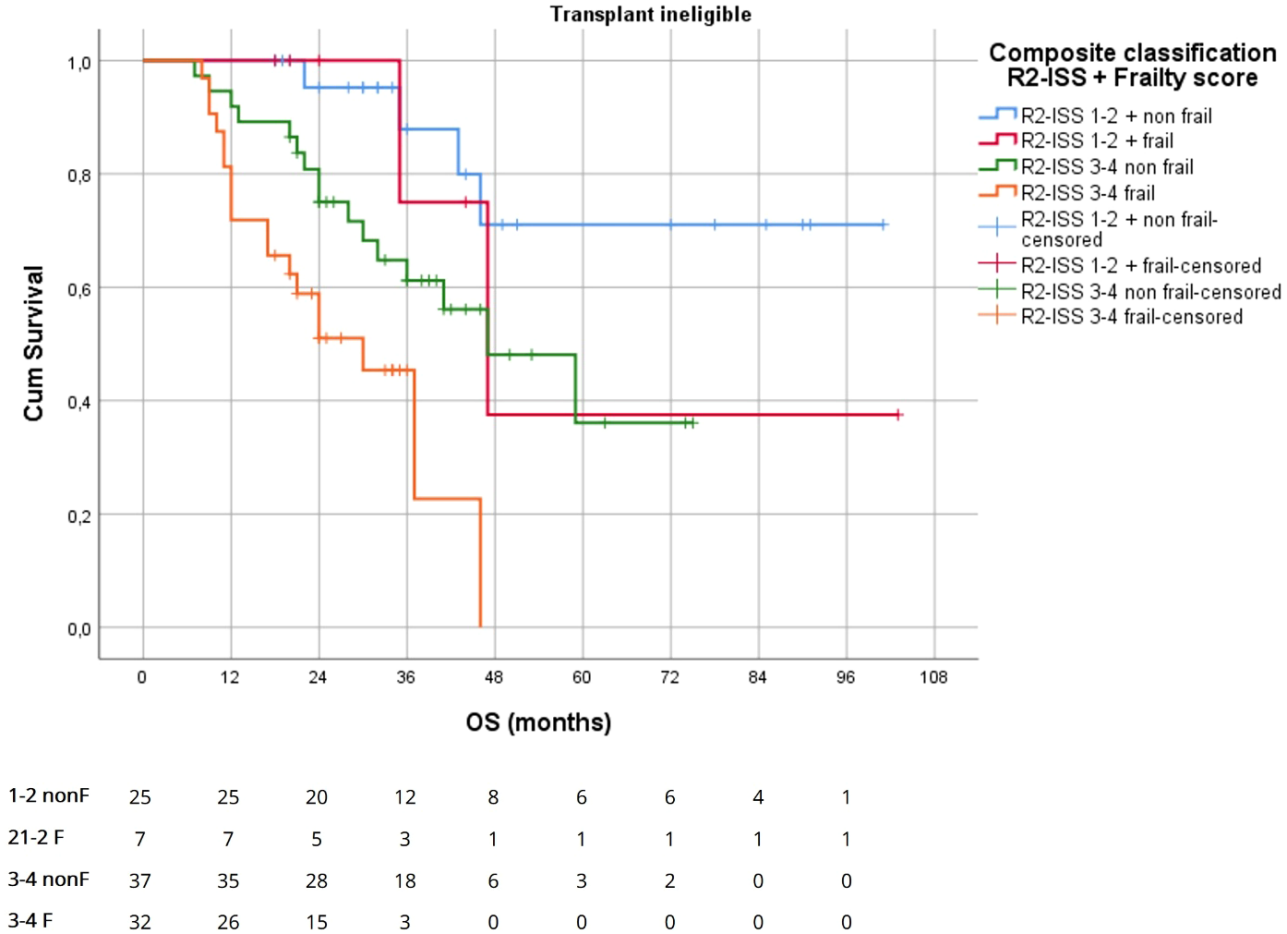
Kaplan–Meier OS sub-analysis according to the composite classification including R2-ISS and IMWG frailty score.

Importantly, the OS analysis of the composite classification according to the frailty status and R2-ISS indicated significant difference (log-rank test: χ² = 21.414, df = 3; p < 0.001) between following categories: R2-ISS 1-2 + non-frail *vs.* R2-ISS 1-2 + frail *vs.* R2-ISS 3-4 + non-frail *vs.* R2-ISS 3-4 + frail (**Figure 6**).

In the multivariate Cox regression model for OS, R2-ISS (HR 1.32, 95% CI 1.07-1.63; p=0.009), IMWG frailty status (HR 2.39, 95% CI 1.80-3.18; p<0.001), and achievement of treatment response (HR 0.39, 95% CI 0.26-0.58; p<0.001) were independently associated with OS. Based on the likelihood-ratio (Δ−2 log likelihood) analysis, the IMWG frailty status most significantly contributed to the OS in the Cox regression model, followed by the treatment response categories and R2-ISS stratification.

## Discussion

Accurate risk stratification NDMM requires consideration of disease-related biological factors as well as patient-related characteristics that may influence treatment response and long-term outcomes. While the R2-ISS represents the current standard for biological risk assessment, frailty status has become as an equally important determinant of treatment response and survival in routine clinical practice (3–5, 15). In the present real-world study, we demonstrated that both the R2-ISS and the original IMWG frailty score independently predicted treatment response and overall survival, while their combined assessment provided additional prognostic discrimination beyond disease-related factors alone.

The distribution of patients across the R2-ISS categories in our cohort was comparable to that reported in the original EMN HARMONY study, supporting the applicability of this staging system in routine clinical practice (3). Importantly, patients with higher R2-ISS stages were significantly less likely to achieve a favorable treatment response, indicating that increasing biological risk adversely affects not only long-term survival but also the probability of achieving an treatment response. Similar observations have been reported in several real-world validation studies from Australia, New Zealand, the Balkan Myeloma Study Group, Japan, Korea, and transplant cohorts, all confirming the reproducibility and prognostic robustness of the R2-ISS across different clinical settings (6–9, 17–19).

Consistent with previous reports, increasing R2-ISS stage was associated with significantly shorter PFS and OS in our cohort. Patients classified as R2-ISS III and IV experienced the poorest outcomes, confirming the ability of the revised staging system to identify patients with aggressive disease biology (3, 6–9, 17–19). These findings further support incorporation of the R2-ISS into routine clinical assessment to facilitate early identification of patients who may benefit from intensified therapeutic strategies, closer monitoring, and enrollment in clinical trials evaluating novel treatment approaches.

An important observation of the present study is that frailty independently influenced both treatment response and survival outcomes. Fit patients achieved significantly higher response rates and experienced substantially longer PFS and OS than intermediate-fit and frail patients. These findings are in accordance with the original IMWG frailty model and subsequent EMN recommendations demonstrating that physiological reserve, comorbidity burden, and functional status substantially influence treatment tolerance and survival independently of disease biology (5, 22). Furthermore, the prognostic significance of frailty remained evident in the subgroup of transplant-ineligible patients, emphasizing that comprehensive geriatric assessment remains clinically relevant even when therapeutic options are limited.

Importantly, multivariable Cox regression analysis demonstrated that R2-ISS, IMWG frailty status, and treatment response categorization were independent factors associated with OS. These findings indicate that biological disease aggressiveness and patient-related vulnerability provide complementary rather than overlapping prognostic information. The independent prognostic value of treatment response is also consistent with previous studies showing that deeper responses are associated with improved long-term survival in MM (20, 21).

One of the most clinically relevant findings of this study is that the composite classification integrating R2-ISS and IMWG frailty score provided additional prognostic discrimination for OS. Patients combining high biological risk (R2-ISS III–IV) with frailty had the poorest survival, whereas biologically lower-risk non-frail patients demonstrated the most favorable outcomes. Notably, frailty further discriminated survival within broader biological risk categories, suggesting that patients with similar disease-related risk may experience substantially different outcomes depending on their functional reserve and vulnerability. These findings support the concept proposed by the EMN that optimal prognostic assessment should integrate disease-related and patient-related variables rather than relying exclusively on biological risk stratification (4, 15, 22).

The treatment landscape of MM has evolved substantially during the study period. Most patients in our cohort received bortezomib-based triplet regimens, which represented the standard frontline treatment at the time of diagnosis. More recently, incorporation of anti-CD38 monoclonal antibodies into induction therapy has significantly improved treatment outcomes across different patient populations, including individuals with high-risk disease (15, 23–26). Whether the prognostic impact of the R2-ISS and frailty assessment will remain unchanged in the era of quadruplet regimens, bispecific antibodies, trispecific antibodies, and chimeric antigen receptor (CAR) T-cell therapy requires further prospective evaluation. Nevertheless, accurate baseline risk stratification is expected to remain essential for treatment selection even as therapeutic options continue to expand.

Our findings should also be interpreted in the light of several limitations. This was a retrospective single-center study reflecting routine clinical practice, which may limit the generalization of the results. In addition, complete cytogenetic information was not available for all patients mirroring real-world circumstances of the routine clinical practice. Yet, only patients with available complete cytogenetic findings were included in the corresponding analyses. Furthermore, treatment strategies evolved during the study period in accordance with contemporary international recommendations. In addition, treatment response is a post-baseline variable and its inclusion in the Cox model without a landmark or time-dependent analysis may introduce potential bias. Accordingly, the association between treatment response and overall survival should be interpreted with caution. However, the relatively large real-world cohort of NDMM patients treated outside of clinical trials in the settings of all of the limitations characterizing routine clinical practice, their comprehensive clinical characterization, and simultaneous evaluation of both biological risk and frailty in consequent treatment decision-making, represent important strengths of the present study.

Overall, our results support the growing concept that prognostic assessment in MM should extend beyond disease biology alone. Integrating the R2-ISS with standardized frailty assessment provides a more comprehensive evaluation of patient prognosis and may facilitate individualized treatment strategies in routine clinical practice. Future prospective multicenter studies incorporating contemporary molecular profiling and novel immunotherapeutic approaches will be important to further refine integrated prognostic models for patients with NDMM.

## Conclusion

The R2-ISS and the IMWG frailty score provide distinct and complementary prognostic information in patients with NDMM. In our real-world cohort, both were independently associated with overall survival, while their combined assessment provided additional prognostic discrimination. Integrating biological disease risk with standardized frailty assessment may enable a more comprehensive evaluation of patient prognosis and support individualized treatment planning in routine clinical practice. Future prospective studies are needed to further evaluate this integrated approach in patients treated with contemporary therapeutic regimens.

## Clinical practice points

The R2-ISS accurately stratifies disease-related risk in patients with newly diagnosed multiple myeloma, while the IMWG frailty score provides complementary information on patient fitness. Combining R2-ISS with the IMWG frailty score improves prognostic discrimination beyond disease biology alone. Integrated assessment of disease risk and frailty may support individualized treatment selection and optimize clinical decision-making in routine practice, particularly for transplant-ineligible patients.

## Data Availability

The raw data supporting the conclusions of this article will be made available by the authors, without undue reservation.
